# Individual perception of bees: Between perceived danger and willingness to protect

**DOI:** 10.1371/journal.pone.0180168

**Published:** 2017-06-29

**Authors:** Mona Lisa Schönfelder, Franz Xaver Bogner

**Affiliations:** Department of Biology Education, University of Bayreuth, Bayreuth, Germany; University of Reading, UNITED KINGDOM

## Abstract

The current loss of biodiversity has found its way into the media. Especially the loss of bees as pollinators has recently received much attention aiming to increase public awareness about the consequence of pollinator loss and strategies for protection. However, pollinating insects like bees often prompt considerable anxiety. Negative emotions such as fear and disgust often lead to lack of support for conservation and appropriate initiatives for protection. Our study monitored perceptions of bees in the contexts of conservation and danger bees possibly represent by applying a semantic differential using contrasting adjectives under the heading “I think bees are…”. Additionally, open questions were applied to examine individual perceptions of danger and conservation of bees. Respondents were students from primary school, secondary school and university. We compared these novices (*n* = 499) to experts (beekeepers, *n* = 153). An exploratory factor analysis of the semantic differential responses yielded three major oblique factors: *Interest*, *Danger* and *Conservation & Usefulness*. The inter-correlations of these factors were significant. Although all subgroups showed an overall high willingness to protect bees, the perception of danger scored medium. The individual experience of bee stings was the most prevalent reason for expressing fear. Educational programs focusing on pollinator conservation may reduce the perceived danger through removing misinformation, and supporting interest in the species. Based on the overall positive attitude toward bees, we suggest introducing bees (e.g., *Apis mellifera*) as a flagship species for pollinator conservation.

## Introduction

Pollination animals are key players in most terrestrial ecosystems, providing an essential ecological service which affects human life directly and indirectly [1,2]. Especially wild and domesticated bees are the primary pollinators of wild plants and agricultural crops. Through their ecological and economic value they hold an exceptional position within global ecosystems [2,3]. Among the generally detected loss of biodiversity [4] there is increasingly strong evidence for a decline in pollinators. This decline constitutes a potential threat to the vital ecological services, and could lead to a lasting negative effect on wild plant diversity, crop production and food security [3]. A variety of possible causes of this documented decline have attracted growing attention in recent decades by the scientific community and general public. A number of studies observed different factors which may be driving the detected loss. Habitat loss, parasites, disease as well as pesticides are the reported major stressors [5]. It should be underlined that in the majority of cases these factors do not act in isolation. Rather the interaction between these factors leads to harm, and this interaction seems to vary in different parts of the world [5]. Striving for a well-balanced healthy planet, awareness of pollinator conservation is needed at the local and global levels [6]. In recent years, various actions, campaigns and programs all over the world have been implemented to raise public awareness of the significance of pollinator conservation [1,7]. In the case of bees, the phenomenon of Colony Collapse Disorder, the unexpected loss of honeybee colonies, has attracted great attention among researchers, politics and the public in recent years [6,8]. One fundamental tool to locally counteract the current trend in biodiversity loss is environmental education [5,9], aiming to foster awareness of the conservation of biodiversity.

Insects and other invertebrates are often associated with negative emotions such as dislike, fear and aversion [10–12]. Attitudes may be described as a complex construct, consisting of cognitive (e.g. knowledge, ideas, thoughts), affective (e.g. emotions, feelings) and conative (e.g. intended behavior) components which strongly influence each other [13]. Negative attitudes toward animals are assumed to be due to a biological predisposition to be prepared for potentially dangerous species [14] in order to defend oneself against predators, or avoid diseases and infections [15,16]. Focusing on the cognitive component, negative perceptions of animals are often accompanied by myths and superstitions [17] as well as by other cultural and/or individual factors [18,19]. Thus, potential alternative conceptions or misinformation, aligned with personal experience, media or formal interventions can influence attitudes [20]. In comparison, fear and disgust as parts of the affective component are based on social learning (instruction and observation) [21] and personal experiences (conditioning) [22,23]. Especially emotional responses toward animals are well documented in the scientific literature (e.g. [24–26]). Previous studies about attitudes toward animals often refer to nine fundamental attitudinal ‘types’: aesthetic, dominionistic, ecologistic, humanistic, moralistic, naturalistic, negativistic, scientistic, and utilistic [27]. These types are influenced by diverse personal variables, such as gender or age [28,29]. For instance, the attitudes of 6 to 9 year-old children toward animals seem to be dependent on affective and emotional influences (e.g. high utilitarian, dominionistic and moralistic scale results) while for 10 to 13 year olds cognitive components (e.g. factual knowledge) seem to be prevalent. 13 to 16 year old students’ attitudes are characterized by an increase in ethical concerns and ecological appreciation [27]. Further studies examining the likeability of different animal species found gender, age and educational level to be predictive for individual preferences [30]. Several studies confirm that vertebrates, especially mammals, are preferred over invertebrates (e.g. [31,32]. Although the fear of wasps and bees seems more intense [31,33], insects with a practical value (e.g. bees) are perceived more positively [11].

As shown by the association of environmental attitudes with pro-animal attitudes [34], the likeability of a species also affects conservation concern [35,36]: people are less willing to protect biodiversity when unpopular species are involved [37]. Additionally, Knight [38] pointed out that the support of species protection is significantly related to attitude types, for instance, aesthetic, moralistic as well as negativistic (e.g. fear). More specific investigations showed that fear and disgust [12,39] but also beliefs in superstitions and myths [40] compromise a person’s willingness to protect species.

Raising awareness about the importance of animal conservation and at the same time fostering pro-environmental behavior is a central issue of educational settings [9,41]. Education should focus on attitudes toward animals [34], as negative emotions could hinder successful learning [42]. Knowledge about peoples’ existing attitudes is essential when educational programs are designed. Emotional perceptions toward unpopular animals can for example be systematically reduced within educational settings [25,43].

Surprisingly, there is a lack of studies on attitudes toward bees, although pollinator conservation seems to hog the limelight in current media and is part of school curricula in Germany and elsewhere. As mentioned before, the association of fear in regard to bees was recently investigated, but often only in combination with wasps [30,33]. Our study aims to explore how people perceive bees, in order to design effective educational programs supporting pollinator conservation. In comparison to most recent studies we use a sematic differential to investigate the perception of bees. Since we compare different age groups of students as well of beekeepers as experts, we hope to respond to all ages through this method. We focus on selected individual aspects of attitudes, namely the perceived danger, the willingness to protect bees and interest. The aim of our study is threefold: First, to investigate whether a semantic differential is an appropriate instrument for measuring the perception of bees regarding the aspects danger, conservation and interest. Second, to examine the relationship between the perception of bees as being dangerous and the willingness to protect them. Third, particularly with regard to design future effective educational programs, to investigate the perception of bees in regard to danger, conservation and interest. We focus on how age, or rather the level of expertise, influences the examined aspects.

## Material and methods

### Ethics statement

The proposed research and consent processes were approved by the Bavarian Ministry of Education (“Bayerisches Staatsministerium für Bildung und Kultus, Wissenschaft und Kunst”) in April 2014 (III.9-5 O 5106/100/11). The permit number allows public review of the questionnaires used in the study. Participating schools were informed about the research conducted and provided their consent. All participants or legal guardians provided their written or oral consent to participate in this study. Data privacy laws were respected as our data was recorded pseudo-anonymously. Only the specific identifier number, based on sex, birth month and year allows conclusions on sex and age. Participants and legal guardians had the chance to reject study participation at any time.

### Participants

Two groups were compared: experts and novices (Table 1). The expert group comprising experienced beekeepers was surveyed at a regional beekeeper convention. The novices consisted of subgroups determined by age and levels of expertise. We examined fourth- and fifth-grade pupils (primary school) and seventh and eighth graders (secondary school). Overall, 15 classes from five different schools participated in our study. All schools are located in major district towns or in suburbs in Bavaria, Germany. Thus, our participants were supposed of growing up in more rural regions rather than big city environments. We also collected data from university students from a variety of disciplines, excluding those with a background in biology to avoid distortions based on the level of expertise. The gender distribution was well balanced except for the beekeepers subgroup, which includes a higher proportion of male participants (Table 1). This may be due to the fact that beekeeping has long been a male domain [44].

**Table 1:** Sample characteristics.

		Age		Gender [%]	
	*n*	*M*	*SD*	male	female
**Novices**					
(1) Pupils (Primary School)	78	10.4	0.7	43.6	56.4
(2) Pupils (Secondary School)	321	13.6	0.7	56.7	43.3
(3) University students	100	22.8	2.4	44.0	56.0
**Experts**					
(4) Beekeepers	153	57.8	13.5	67.6	32.4

*N* = 652

### Instruments

A paper-pencil-test was applied using semantic differential and open questions to collect attitudes and ideas about bees. Semantic differentials measure attitudes by asking participants to position themselves between two polar adjectives [45]. Based on adjectives adopted from Drissner et al. [46], participants were requested to position themselves on a nine-point scale between eight word pairs (e.g. “dangerous-safe”, “fascinating-boring”, or “valuable-useless”) in reference to the statement “I think bees are…“. Attributes were chosen focusing on danger, utilization, conservation and interest toward bees. For a better understanding of the ideas behind participants’ attitudes toward perceived danger and willingness to protect bees, two additional open questions were applied to all participants: “Explain why bees are supposed to be dangerous/safe in your opinion?” and “explain why bees are supposed to be worthless/worth to be protected in your opinion?”. Predefined lines supported participants for the expected statement length.

### Data analyses

Statistical tests were conducted in SPSS (Version 22.0). All analyses were based on non-parametric tests due to a partially non-normal distribution of variables.

The factor structure of the semantic differential was extracted using an exploratory principal-axis factor analysis. Oblique rotation was applied [47]. The following tests were applied using factor scores, taking the dimension of single factor loadings into account. A bivariate correlation of the detected factors was calculated.

A comparison of subgroups within each factor was calculated using Kruskal-Wallis tests and pair-wise post-hoc analyses based on Mann-Whitney-U tests. Performing multiple tests we avoided cumulative Type I errors through a Bonferroni correction [48]. According to Field [47] we calculated the effect size *r*, whereby effects are interpreted as .10 ‘small effect’, .30 ‘medium effect’ and .50 ‘large effect’ [49].

Qualitative content analysis was used to assess the answers we received for our open questions [50]. Based on the expert responses, we inductively built four categories with eight subcategories on the question about perceived danger (Coding guidelines, see S1 Table) and four categories and nine subcategories on the question about the willingness to protect bees (S2 Table). The novice responses were assigned deductively to the subcategories according to our coding guidelines. A person’s statement could be classified into several categories.

To ensure the reliability of our categorization we randomly selected about 15% of all novice and expert answers. The analysis of inter- and intra-rater reliability, using Cohen’s kappa coefficient [51], yielded scores between .84 and 1, reflecting an ‘almost perfect’ consistency of category assignment (Table 2) [52].

**Table 2:** Cohen’s kappa scores for inter- and intra-reliability.

	Cohen’s kappa	
	Inter-rater-reliability	Intra-rater-reliability
**dangerous vs. safe**		
experts	.90	.95
novices	.91	.93
**worthless vs. worth to be protected**		
experts	.91	1
novices	.84	.96

We identified categories for perceived danger and willingness to protect bees, and calculated the frequency of their occurrence. The differences between subgroups were analyzed using Pearson’s chi square tests. We calculated the adjusted contingency coefficient *C* whose range extends from 0 to 1.

## Results

### Factor structure of the semantic differential

The principal-axis factor analysis reduced the initial eight sematic differential pairs to three factors (based on the eigenvalue criterion surpassing 1). Items clustering under the same factor can be interpreted as follows: *Interest*, *danger* and *conservation & usefulness*. *Interest* and *conservation & usefulness* consisted of three word pairs each and *danger* of two word pairs. The Kaiser-Meyer-Olkin measure confirmed the sampling adequacy for the factor analysis with a ‘middling’ KMO value of all items (.79) according to Hutcheson and Sofroniou [53] and values for individual items greater than .61, which pass the acceptable limit of .5 [47]. Altogether, the three extracted factors explained 67.10% of the total variance. Table 3 displays the factor scores after rotation as well as the internal consistency (Cronbach’s alpha) for the single factors as predictor for reliability.

**Table 3:** Exploratory factor analysis of the semantic differential.

Factor	Item	Factor Loadings			Eigen value	Cronbach’s α value
		INT	DANG	CON		
*INT*	*Interest*				3.88	.87
INT1	fascinating—boring	.95				
INT2	interesting—uninteresting	.87				
INT3	cool–uncool	.49				
*DANG*	*Danger*				1.33	.82
DANG1	harmless—weird		.91			
DANG2	safe—dangerous		.78			
*CON*	*Conservation & Usefulness*				1.09	.79
CON1	valuable—useless			.80		
CON2	necessary—unnecessary			.73		
CON3	worth protecting—worthless			.71		

Factor loadings below .40 are omitted; *N* = 511.

*Interest* and *danger* correlated negatively and significantly with a medium effect size (*r*_s_ = -.41, 95% BCa CI {-.48, -.32}, *p* < .001). A larger effect was found for the correlation of *interest* with *conservation & usefulness* (*r*_s_ = .69 {.63, .74}, *p* < .001) as well as for *danger* with *conservation & usefulness* (*r*_s_ = -.52 {-.59, -.45}, *p* < .001).

### Subgroups’ perceptions of bees

**Fig 1:**
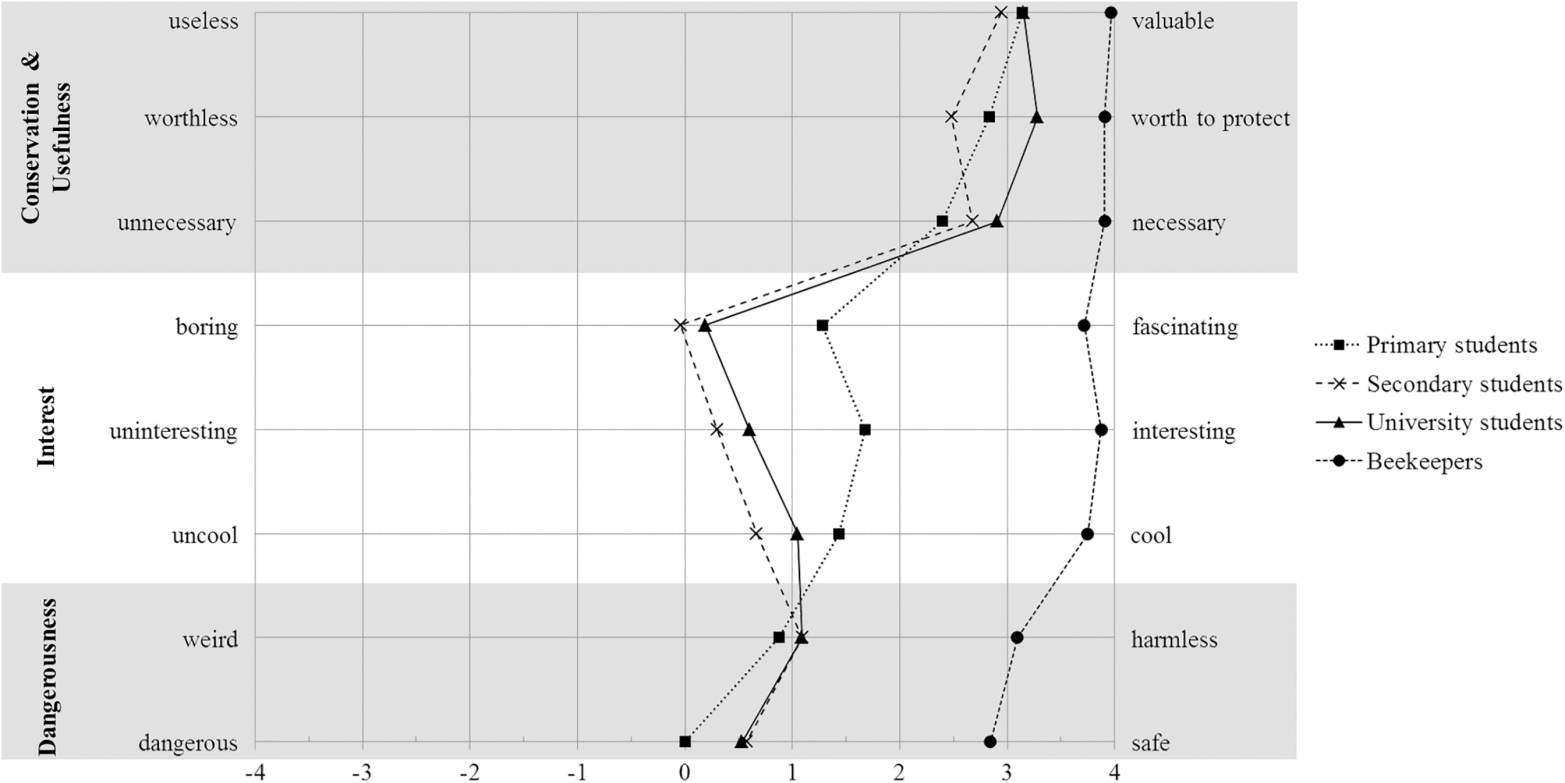
Attitudes toward bees: Subgroup profiles. Related word pairs of the semantic differential to be found left and right of the diagram. Adjectives reflecting a positive attitude toward bees are place on the right side.

Participants’ perception of bees was investigated by applying the semantic differential. In general, individual ratings were shifted toward the positive adjective of a word pair. Expert scores in comparison to novice scores reflect a very positive attitude toward bees (Fig 1). Attitude scores differed significantly between the novice subgroups (*interest*: *H*(3) = 101.26, *p* < .001; *danger*: *H*(3) = 51.12, *p* < .001; *conservation & usefulness*: *H*(3) = 78.92, *p* < .001).

A pairwise post-hoc comparison between all subgroups was calculated to detect differences between the subgroups’ attitudes toward bees (Table 4). The beekeeping experts show a significantly higher *interest* in bees compared to the novice groups. Primary school students show a significantly higher *interest* in bees compared to secondary and university students, but both older groups do not differ from each other. Concerning the perceived *danger* of bees, the novice subgroups do not differ from each other, but perceive significantly more danger (medium to large effect size) than beekeepers. All subgroups perceived bees to be useful and worthy of conservation (*conservation & usefulness*). However, as experts also differ significantly from novices, the novice subgroups only showed a significant difference between secondary school and university students, only with a small effect

**Table 4:** Pairwise comparison of subgroups for the factors *Interest*, *Danger* and *Conservation & Usefulness* including a summary of subgroup medians and interquartiles.

			Subsamples											
			prim.			sec.			univ.			beek.		
	Mdn	IQR	*U*	*p*	*r*	*U*	*p*	*r*	*U*	*p*	*r*	*U*	*p*	*r*
**Interest**														
prim.	0.43	1.48	-	-	-	7079.00	< .001^**^	-.25	2677.50	.002^*^	-.23	211.50	< .001^**^	-.66
sec.	-0.20	1.15	-	-	-	-	-	-	13666.00	.098	-.08	283.00	< .001^**^	-.49
univ.	0.08	1.38	-	-	-	-	-	-	-	-	-	84.00	< .001^**^	-.71
beek.	1.58	0.12	13666.0-	-	-	13666.0-	-	-	-	< .01^**^-	-	13666.0-	-	-
**Danger**														
prim.	0.04	1.42	-	-	-	10598.50	.552	-.03	3469.50	.508	-.05	324.50	< .001^**^	-.58
sec.	0.13	1.25	-	-	-	-	-	-	15294.00	.955	-.03	1350.00	< .001^**^	-.38
univ.	-0.00	1.23	-	-	-	-	-	-	-	-	-	368.00	< .001^**^	-.58
beek.	-1.18	0.68	-	-	-	-	-	-	-	-	-	-	-	-
**Conservation & Usefulness**														
prim.	0.18	1.31	-	-	-	3583.50	.753	-.02	9577.50	.069	-.09	222.50	< .001^**^	-.65
sec.	0.03	1.24	-	-	-	-	-	-	12614.00	.007^*^	-.13	523.00	< .001^**^	-.46
univ.	0.37	0.90	-	-	-	-	-	-	-	-	-	232.00	< .001^**^	-.64
beek.	0.96	0.06	-	< .01^**^-	.25-	-	-	-	-	-	-	-	-	-

Mann-Whitney test *U*; after Bonferroni correction: p^*^significant at *α* < .008 and p^**^significant at *α* < .002; effect size *r (*$r=z/\sqrt{N}$*)*

### Reasons for perceived danger of bees

The qualitative content analysis revealed participants’ ideas about the danger and conservation of bees. Most of the reasons concerning danger were conditional. For instance, participants mentioned that bees in general are safe, but have the potential to be dangerous (“Bees just defend their bee colony, otherwise they are safe”). The most frequent reasons mentioned for perceived danger were grouped into the categories *character of bees*, *bee sting* and *handling of bees* (Table 5).

**Table 5:** Choice of individual reasons for dangerousness and conservation.

Reasons (answers in %)	prim.^a^	sec.^b^	univ.^c^	beek.^d^
***dangerous vs*. *safe***				
Character of bees	18.4	24.7	35.2	56.2
Bee sting	72.4	65.9	58.2	28.6
Handling of bees	39.5	38.6	29.7	26.7
***worthless vs*. *worth to be protect***				
Bee products	59.7	38.6	39.1	18.6
Pollination (in general)	44.4	54.9	59.8	78.8
*Importance* of pollination for humanity	12.5	19.0	19.6	29.2
Ecological importance of pollination	8.3	19.9	44.6	41.6
Extinction of humanity	2.8	21.6	5.4	6.2

A participant’s answer can be assigned to multiple categories

^a^*n* = 76

^b^*n* = 308

^c^*n* = 92

^d^*n* = 113

We conducted contingency analyses in order to see if the frequency of the mentioned reasons is significant associated to the level of expertise. In the case of the *character of bees* a significant association to the level of expertise exists (χ^2^(3) = 43.10; *p* < .001; *C*_*corr*_ = .33). Most of the experts (56.2%) mentioned that the danger of bees is connected to their character, (e.g. specific behavior like hive defense) arguing from the bees’ perspective and 18.1% of the beekeepers explicitly refer to breeding a peaceful race. The number of experts mentioning the bee character as potential reason for its danger is significantly higher than the number of novices mentioning the bee character (univ. vs. beek.: χ^2^(1) = 8.67; *p* = .003; *C*_*corr*_ = .29). Although the novice groups indicated a clear trend showing that older novices mention the bees’ character more often than the younger novices, these differences were not significant (*p* significant at α < .008 after Bonferroni correction).

Equally, we found a significant association in the category *bee sting* (χ^2^(3) = 51.82; *p* < .001; *C*_*corr*_ = .36). While the frequency of mentioning *bee sting* tended to decline with decreasing age, the novice groups did not significantly differ from each other. Only the beekeepers (28.6%) mentioned *bee sting* less frequently than the novice groups (univ. vs. beek.: χ^2^(1) = 17.58; *p* < .001; *C*_*corr*_ = .41). Nevertheless, the bee sting is the most common reason mentioned by all groups of novices, sometimes commenting that the bee sting is generally problematic (prim. 13.2%, sec. 6.8%, univ. 6.6%, beek. 1.0%) or problematic especially for persons with bee venom allergy (prim. 5.3%, sec. 13.6%, univ. 14.3%, beek. 20.0%). Only few participants commented that the bee sting is unproblematic (prim. 2.6%, sec. 8.4%, univ. 8.8%, beek. 5.7%).

The category *handling of bees* summarizes all active human behavior mentioned regarding the handling of bees (e.g. to provoke bees). Within this category the contingency analysis did not detect differences between the subgroups concerning the frequency of mentioning this reason. Nonetheless, with the level of expertise mentioning *handling of bees* tended to decrease.

### Reasons for the willingness to protect bees

On the willingness to protect bees, almost all participants have the same opinion, which led us to categories dealing with reasons why bees are worth to be protected (prim. 97.2%, sec. 95.4%, univ. 98.9%, beek. 100%). The most frequently mentioned reasons were *bee products*, *pollination* in general, *importance of pollination for humanity* and *ecological importance of pollination* and *extinction of humanity* (Table 5).

Mentioning *bee products* as a reason for conservation is significantly associated with the level of expertise (χ^2^(3) = 32.71; *p* < .001; *C*_*corr*_ = .29). Most of the primary school students (59.7%) mentioned products like honey, wax, etc. as reason for protection, which differs from older students (prim. vs. sec.: χ^2^(1) = 10.67; *p* = .001; *C*_*corr*_ = .26). In general, experts mentioned bee products less frequently as a reason for conservation (beek. vs. sec.: χ^2^(1) = 14.86; *p* < .001; *C*_*corr*_ = .23) but pointed to *pollination* as major reason. We found an association between the frequency of mentioning pollination and the level of expertise (χ^2^(3) = 26.70; *p* < .001; *C*_*corr*_ = .27). Although the frequencies suggested an increasing trend along the level of expertise, the novice groups did not differ from each other significantly, only the experts (beek. vs. univ.: χ^2^(1) = 8.74; *p* = .003; *C*_*corr*_ = .29).

We counted the frequencies of mentioning pollination in general as well as the more precise statements about the importance for humanity or the ecosystem. The frequency of mentioning the *importance for humanity*, such as being able to harvest fruits or crops, is not associated to subgroups. More than 10% of the participants mentioned the importance of pollination services for humanity, whereas with age and level of expertise the importance for humanity is mentioned more often. The frequency of mentioning the *ecological importance of pollination*, however, seems distributed (χ^2^(3) = 47.50; *p* < .001; *C*_*corr*_ = .35). The subgroups are split into two clusters: the primary and secondary school students (prim. 8.3%, sec. 19.9%) and the university students and beekeepers (univ. 44.6%, beek. 41.6%) differ significantly from each other (sec. vs. beek.: χ^2^(1) = 20.23; *p* < .001; *C*_*corr*_ = .30). The latter group answered more than twice as frequently with reasons like the importance for an ecological balance or the conservation of biodiversity.

Surprisingly, we derived one category including all answers related to an *extinction of humanity*. Respondents often referred to a quote which is erroneously attributed to Albert Einstein [54] (p. 34) or their answers contained explanations about the reduction of oxygen if the bee as a pollinator would go extinct. The frequencies of mentioning the extinction of humanity as reason for the conserving of bees are not distributed as expected (χ^2^(3) = 33.94; *p* < .001; *C*_*corr*_ = .30). The secondary school students form a distinct subgroup as they mention the extinction of humanity most often (sec. vs. beek.: χ^2^(1) = 13.56; *p* < .001; *C*_*corr*_ = .25) with every fifth student mentioning the extinction of humanity as reason why bees are worth to be protected.

## Discussion

Against the background of running into danger of a biodiversity loss of important pollinators, it is crucial to better understand people’s attitude toward selected species [38]. Bees as most prominent pollinators are ubiquitous in current media and school curricula. However, there is a lack of studies investigating peoples’ perception of bees. The present study monitored attitudes toward bees from novices and experts regarding the perceived danger and the willingness to protect them, and also examined qualitatively collected data to understand the reasons behind the gathered perceptions.

### Factors influencing the willingness to protect bees

Negative perceptions of animals are supposed to interact with individual conservation efforts [37,38]. We also found a significant relationship between the perceived danger and the willingness to protect bees. This result matches previous studies dealing with a negative influence on support of a perceived danger [12] as well as fear and disgust as emotions toward different animals [38,55]. Next to a perceived danger which affects peoples’ willingness to protect, we also detected a significant correlation between willingness to protect and interest. We assume that participants displaying a more positive attitude toward bee protection are generally more interested in bees. This relationship agrees with the study of Lindemann‐Matthies [56] who concludes that raising students’ interest in animals represents an important contribution to their attitude toward conservation. This effect has recently been demonstrated by Ballouard et al. [43] who implemented an educational program and observed the reduction of fear and the increase of willingness to protect even unpopular animals, like snakes. To enhance peoples’ willingness to protect bees, it seems crucial to consider their perception of danger as well as their interest in the species.

### Novices’ and experts’ attitudes toward bees

Our novice subgroups (primary, secondary and university students) show an overall positive attitude toward bees. The beekeepers, as expected, show even stronger positive attitudes toward bees (ceiling effect) concerning all three attitude aspects: interest, perceived danger and the willingness to protect bees. Thus, we regard our experts as a reference in our present study. The novice subgroups do not differ significantly from each other in their rating of danger and conservation & usefulness, but they differ concerning their interest in bees. In our study, primary school students show the greatest interest, which is again in line with earlier studies. Younger students are more interested in biological topics in general [57,58], and in living organism in particular [56] compared to older students. It is also conceivable that young student’s interest in such topics is reflected in their connectedness to nature: Younger children feel also more connected to nature [59] and express more pro-environmental attitudes [60].

### Why do people consider bees to be dangerous?

Our participants perceived the danger of bees as lower although earlier studies had found bees and some taxonomically related species (wasps, hornets) as perceived dangerous [31,33]. In our case both novices and experts referred to a conditional danger: participants, for instance, mentioned that bees are not a threat unless they are provoked. Most associations of novices regarding perceived danger explicitly dealt with bee stings. This result can be compared to research literature dealing with the human fear of arthropods [33]. Obviously, the sting is the crucial factor for a perceived danger of hornets, wasps and bees are considered the most dangerous arthropods [22,61]. Not surprisingly, fear and connected negative emotions are impacted by prior experience and knowledge [23] which is reflected in our findings: Beekeepers did not mention stings as most crucial factor for conditional danger, although they may get stung more often than lay people. Novices do not have as much experience with bees as beekeepers do, and children and adolescents may obviously have negative emotions toward getting stung by bees (or other hymenoptera). Experiences of pain and swelling associated with stings from insects as well as the knowledge of existing bee venom allergies may be causes of novices’ perceived danger within this context [10].

Other stated reasons for a perceived danger are the *character of bees* and the *handling of bees*. It is conspicuous that in connection with danger the *character of bees* is stated more often increasing with the expert level while *handling of bees* tends to decrease with the expert level. This fact implies a shift in focus of the argumentation from the human (handling of bees) to the bees’ perspective (character of bees) along the expert level. Both perspectives obviously are influencing each other and additionally provide information about different ways of thinking about the perceived conditional danger. A more egocentric perspective found in the younger students (primary and secondary school) may also be based on differences between children and adults regarding the ability to change perspective. Adults (beekeepers and university students) tend to be less egocentric than children [62].

Concerning bee stings, it is difficult to clearly tell whether novices only refer to bees, or if they also refer to other insects which look similar to bees, like wasps or hornets. Prior studies have shown that people rate some species as fear-relevant because of misidentifying e.g. hoverflies or bumblebees for bees [22] which indicates a lack of knowledge of species. Educational programs should, therefore, focus on the following two aspects to reduce the perceived danger of bees for humans: (1) the special character of bees, or rather their breed and their behavior and (2) the ability to differentiate bees from insects with a similar appearance (e.g. wasps).

### Why do people think bees are worthy of protection?

The remarkable positive perception of bees concerning conservation and usefulness in all groups was surprising, which we consider as a high willingness to protect bees. Although age affects environmental attitudes and awareness in general [63] we could not show significant age differences in the attitude toward the conservation of bees. However, the reasons why students and beekeepers think bees are worth protection are of specific interest: Particularly young students frequently stated bee products as a crucial factor for protecting bees rather than the pollination services, the most frequently stated argument of all other participants. This finding is consistent with Kellert’s study [27] where the utilitarian attitude toward animals decreased and the ecological attitude increased between the 2^nd^ and the 11^th^ grade. In our opinion, these results also reflect the continuing lack of understanding of the abstract ecological concept primary school students hold [64,65] and the egocentric view of children [62]. Knowing about pollination is generally due to individual experience and/or educational efforts and should increase naturally with age and expert level.

Although pollination is the most stated argument in total, the subgroups still differ in the specification of their answers: While primary school students rarely mentioned the pollination service, university students and beekeepers in particular highlight the ecological importance as a major reason for protecting bees. Due to the media, the current losses of honeybee colonies raised great attention [8] and informed the public about the importance of animals’ pollination services. Beside the experts, who naturally show a great interest in bees, especially older students may come into contact with media-present socio-ecological issues and may, therefore, be more sensitized toward pollinator conservation.

Interestingly, about every fifth secondary school student believes that bees need protection because humanity would die out if bees became extinct. This is an association which can be regarded as an alternative conception. Such conceptions can be described as “any conceptual difficulties, which is different from or inconsistent with the accepted scientific definition” [66]. Often respondents explicitly referred to a quotation of Albert Einstein’s: “If the bee disappeared off the surface of the globe, then man would have only four years of life left. No more bees, no more pollination, no more plants, no more animals, no more man” [54] (p.34). While this sentence is often already used for honeybee conservation and seems to be well-known, no evidence is traceable that he ever pronounced this. It is assumed that activists attributed this sentence to Einstein in order to give the issue more credibility [54]. Although this quotation points to the importance of the species for our lives and the whole ecosystem, it is controversial because of the lack of scientific accuracy [67]. The statement about the extinction of humanity frequently appeared in the answers of secondary school students. Due to the small number of surveyed schools we assume that many secondary school respondents were classmates and thus teaching in school promoted this alternative conception. In future educational programs, a scientifically correct content should be ensured: For instance, if bees went extinct, food production would be affected and decline, but nevertheless still exist. The human race would not face extinction because the general pollination of plants is still assured through other pollination mechanisms, such as anemophily. Instead of only focusing on honeybees as pollinators, the topic offers the possibility to stimulate learners to think about effects of environmental conditions on the plant-pollinator interaction. Hence, honeybees would function as an exemplary species to explain the functionality of ecosystems in a broader context.

### Methodological aspects

This study presented a short, valid and reliable instrument to measure individuals’ perceptions of bees concerning different aspects. We based a semantic differential on eight word pairs clustered into three factors. Whereas the detected factor *danger* obviously describes the perceived danger of bees, the factor *conservation & usefulness* summarizes willingness to protect bees, associated with the perception of the animal’s usefulness. Moreover, the factor *interest* describes a general interest in bees. Evidence for the content validity is displayed by comparing experts and novices in Table 4. The beekeepers with more contact, knowledge and experience with bees, also showed a significantly higher interest, a lower perceived danger and a higher willingness to protect bees. This result is underlined by the answers given to the open questions in which the experts showed a significantly different response pattern. Furthermore, the overall internal consistency, shown by Cronbach’s alpha, is good (≥.79).

One limitation of our study is that we just concentrated on students’ and beekeepers’ perceptions of bees. Since we focus on designing effective educational programs on pollinator conservation in formal learning settings, knowledge about students’ attitudes is crucial. We explicitly used a potential bias of beekeepers having an enormously positive attitude towards bees in order to validate our instrument (content validity) and to get a reference that peoples’ attitudes could be further improved.

Not only formal learning settings should be used to raise awareness for pollinator conservation, but also all levels of education, such as informal education and other initiatives should be addressed [7]. Therefore, our validated instrument could be applied in further studies to gather data about attitudes towards bees or other pollinators. For instance, it would be interesting and substantial getting aware of a general societies’ attitude towards bees or of other specific groups such as farmers.

## Conclusions

This study is the first one to focus on peoples’ attitude toward bees. Considering the current and pressing need to conserve pollinating animals, it is crucial for educators to be aware of attitudes toward animals like bees. We found that perceived danger, interest and the willingness to protect bees are interrelated. Therefore, reducing fear and simultaneously increasing interest could be key aspects in educational settings. As the topic “bees as social insects” and “pollination” are part of nearly all trans-national curricula, we strongly suggest connecting both issues and additionally consider the following aspects:

First, we recommend a learning approach with an affective focus, since negative emotions like disgust and fear can be reduced by encountering original objects [25,68]. Generally, encounters with nature foster feelings of connectedness to nature, which in turn can affect the willingness to protect nature [69]. Therefore, we recommend learning programs or interventions where students are brought into contact with living animals. Nevertheless, forcing people with greater fear to handle or touch animals against their might miss the intent and produce the contrary.

Second, we would like to emphasize the need to teach species identification skills, so that different hymenoptera genera and species can be differentiated. Thus, experienced insect stings could be attributed to the responsible species, and hence counteract misattribution.

Third, we recommend focusing on scientifically correct contents in classroom to counteract alternative conceptions. People should understand ecological interrelations and be aware of the key position held by pollinating animals.

Our study found that people show more positive attitudes toward conservation of bees than we would have expected. Besides being quite popular, bees also meet all criteria to be selected as flagship species as described by Schlegel et al.[70]: According to their criteria, bees (i) are local species in most parts of the world [71], (ii) are ecological key players because of their pollinating service [2], (iii) should be identified for example because of their prominence in most educational curricula and current media, (iv) but are not explicitly used as flagship species yet, (v) have a familiar name which is known across all ages and levels of expertise. Consequently, we strongly propose using bees, for instance *Apis mellifera*, as a flagship species for local conservation of pollinating insects.

## Supporting information

S1 Table Inductively built categories for the open question “Explain why bees are supposed to be dangerous / safe in your opinion?”. (DOCX)

S2 Table Inductively built categories for the open question “Explain why bees are supposed to be worth protecting / worthless in your opinion?”. (DOCX)

## References

[1] Kearns CA, Inouye DW, Waser NM. Endangered mutualisms: The conservation of plant-pollinator interactions. Annu Rev Ecol Syst. 1998;29: 83–112. doi: 10.1146/annurev.ecolsys.29.1.83

[2] Losey JE, Vaughn M. The economic value of ecological services provided by insects. Bioscience. 2006;56: 311 doi: 10.1641/0006-3568(2006)56[311:TEVOES]2.0.CO;2

[3] Potts SG, Biesmeijer JC, Kremen C, Neumann P, Schweiger O, Kunin WE. Global pollinator declines: Trends, impacts and drivers. Trends Ecol Evol. Elsevier Ltd; 2010;25: 345–353. doi: 10.1016/j.tree.2010.01.007 20188434 10.1016/j.tree.2010.01.007

[4] Díaz S, Fargione J, Chapin FS, Tilman D. Biodiversity loss threatens human well-being. PLoS Biol. 2006;4: e277 doi: 10.1371/journal.pbio.0040277 16895442 10.1371/journal.pbio.0040277 PMC1543691

[5] Goulson D, Nicholls E, Botias C, Rotheray EL. Bee declines driven by combined stress from parasites, pesticides, and lack of flowers. Science (80-). 2015;347: 1255957–1255957. doi: 10.1126/science.1255957 25721506 10.1126/science.1255957

[6] Byrne A, Fitzpatrick Ú. Bee conservation policy at the global, regional and national levels. Apidologie. 2009;40: 194–210. Available: http://www.scopus.com/inward/record.url?eid=2-s2.0-68349099933&partnerID=40&md5=b0eb28739e81e7ec4a3a680159ab40d7

[7] Abrol DP. Pollination Biology: Biodiversity conservation and agricultural production. Pollinat Biol Biodivers Conserv Agric Prod. 2011; 1–792. doi: 10.1007/978-94-007-1942-2

[8] Neumann P, Carreck N. Honey bee colony losses. J Apic Res. 2010;49: 1 doi: 10.3896/IBRA.1.49.1.01

[9] Brewer C. Translating data into meaning: Education in conservation biology. Conserv Biol. 2006;20: 689–691. doi: 10.1111/j.1523-1739.2006.00467.x 16909556 10.1111/j.1523-1739.2006.00467.x

[10] Davey GCL. Self-reported fears to common indigenous animals in an adult UK population: The role of disgust sensitivity. Br J Psychol. 1994;85: 541–554. doi: 10.1111/j.2044-8295.1994.tb02540.x 7812671 10.1111/j.2044-8295.1994.tb02540.x

[11] Kellert SR. Values and perceptions of invertebrates. Conserv Biol. 1993;7: 845–855. doi: 10.1046/j.1523-1739.1993.07040845.x

[12] Prokop P, Fančovičová J. Does colour matter? The influence of animal warning coloration on human emotions and willingness to protect them. Anim Conserv. 2013;16: 458–466. doi: 10.1111/acv.12014

[13] Eagly AH, Chaiken S. The Psychology of Attitudes. Hartcourt Brace Jovanovich College Publishers; 1993.

[14] Seligman MEP. Phobias and preparedness. Behav Ther. 1971;2: 307–320. doi: 10.1016/S0005-7894(71)80064-3

[15] Davey GCL. Characteristics of individuals with fear of spiders. Anxiety Res. 1991;4: 299–314. doi: 10.1080/08917779208248798

[16] Öhman A, Mineka S. Fears, phobias, and preparedness: Toward an evolved module of fear and fear learning. Psychol Rev. 2001;108: 483–522. doi: 10.1037/0033-295X.108.3.483 11488376 10.1037/0033-295x.108.3.483

[17] Prokop P, Fančovičová J, Kubiatko M. Vampires are still alive: Slovakian students’ attitudes toward bats. Anthrozoos A Multidiscip J Interact People Anim. 2009;22: 19–30. doi: 10.2752/175303708X390446

[18] Herzog HA, Burghardt GM. Attitudes toward animals: Origins and diversity. Anthrozoos A Multidiscip J Interact People Anim. 1988;1: 214–222. doi: 10.2752/089279388787058317

[19] Serpell JA. Factors influencing human attitudes to animals and their welfare. Anim Welf. 2004;13: 145–151.

[20] Mintzes JJ, Wandersee JH. Reform and Innovation in Science Teaching. Teaching Science for Understanding. Elsevier; 2005 pp. 29–58. doi: 10.1016/B978-012498360-1/50003-9

[21] Olsson A, Phelps EA. Social learning of fear. Nat Neurosci. 2007;10: 1095–1102. doi: 10.1038/nn1968 17726475 10.1038/nn1968

[22] Breuer GB, Schlegel J, Kauf P, Rupf R. The importance of being colorful and able to fly: Interpretation and implications of children’s statements on selected insects and other invertebrates. Int J Sci Educ. 2015;0693: 1–24. doi: 10.1080/09500693.2015.1099171

[23] Rachman S. The conditioning theory of fear acquisition: A critical examination. Behav Res Ther. 1977;15: 375–387. 612338 10.1016/0005-7967(77)90041-9

[24] Prokop P, Özel M, Uşak M. Cross-cultural comparison of student attitudes toward snakes. Soc Anim. 2009;17: 224–240. doi: 10.1163/156853009X445398

[25] Randler C, Hummel E, Prokop P. Practical work at school reduces disgust and fear of unpopular animals. Soc Anim. 2012;20: 61–74. doi: 10.1163/156853012X614369

[26] Tomažič I. Seventh graders’ direct experience with, and feelings toward, amphibians and some other nonhuman animals. Soc Anim. 2011;19: 225–247. doi: 10.1163/156853011X578901

[27] Kellert SR. Attitudes toward animals: Age-related development among children. J Environ Educ. 1985;16: 29–39. doi: 10.1080/00958964.1985.9942709

[28] Bjerke T, Ødegårdstuen TS, Kaltenborn BP. Attitudes toward animals among Norwegian adolescents. Anthrozoos A Multidiscip J Interact People Anim. 1998;11: 79–86. doi: 10.2752/089279398787000742

[29] Kellert SR, Berry JK. Attitudes, knowledge, and behaviors toward wildlife as affected by gender. Wildl Soc Bull. 1987;15: 363–371. doi: 10.2307/3782542

[30] Bjerke T, Østdahl T. Animal-related attitudes and activities in an urban population. Anthrozoos A Multidiscip J Interact People Anim. 2004;17: 109–129. doi: 10.2752/089279304786991783

[31] Arrindell WA. Phobic dimensions: IV. The structure of animal fears. Behav Res Ther. 2000;38: 509–530. doi: 10.1016/S0005-7967(99)00097-2 10816909 10.1016/s0005-7967(99)00097-2

[32] Bjerke T, Ødegårdstuen TS, Kaltenborn BP. Attitudes toward animals among Norwegian children and adolescents: Species preferences. Anthrozoos A Multidiscip J Interact People Anim. 1998;11: 227–235. doi: 10.2752/089279398787000544

[33] Gerdes ABM, Uhl G, Alpers GW. Spiders are special: fear and disgust evoked by pictures of arthropods. Evol Hum Behav. Elsevier Inc.; 2009;30: 66–73. doi: 10.1016/j.evolhumbehav.2008.08.005

[34] Binngießer J, Randler C. Association of the environmental attitudes “preservation” and “utilization” with pro-animal attitudes. Int J Environ Sci Educ. 2015;10: 477–492. doi: 10.12973/ijese.2015.255a

[35] Ballouard J-M, Ajtic R, Balint H, Brito JC, Crnobrnja-Isailovic J, Desmonts D, et al Schoolchildren and one of the most unpopular animals: Are they ready to protect snakes? Anthrozoos A Multidiscip J Interact People Anim. 2013;26: 93–109. doi: 10.2752/175303713X13534238631560

[36] Wilson C, Tisdell C. What role does knowledge of wildlife play in providing support for species’ conservation? J Soc Sci. 2005;1: 47–51. doi: 10.3844/jssp.2005.47.51

[37] Martín-López B, Montes C, Benayas J. The non-economic motives behind the willingness to pay for biodiversity conservation. Biol Conserv. 2007;139: 67–82. doi: 10.1016/j.biocon.2007.06.005

[38] Knight AJ. “Bats, snakes and spiders, Oh my!” How aesthetic and negativistic attitudes, and other concepts predict support for species protection. J Environ Psychol. 2008;28: 94–103. doi: 10.1016/j.jenvp.2007.10.001

[39] Prokop P, Fančovičová J. Perceived body condition is associated with fear of a large carnivore predator in humans. Ann Zool Fennici. 2010;47: 417–425. doi: 10.5735/086.047.0606

[40] Ceríaco LM, Marques MP, Madeira NC, Vila-Viçosa CM, Mendes P. Folklore and traditional ecological knowledge of geckos in Southern Portugal: implications for conservation and science. J Ethnobiol Ethnomed. 2011;7: 26 doi: 10.1186/1746-4269-7-26 21892925 10.1186/1746-4269-7-26 PMC3180245

[41] Bogner FX. The influence of short-term outdoor ecology education on long-term variables of environmental perspective. J Environ Educ. 1998;29: 17–29. doi: 10.1080/00958969809599124

[42] Bixler RD, Floyd MF. Hands on or hands off? Disgust sensitivity and preference for environmental education activities. J Environ Educ. 1999;30: 4–11. doi: 10.1080/00958969909601871

[43] Ballouard J-M, Provost G, Barré D, Bonnet X. Influence of a field trip on the attitude of schoolchildren toward unpopular organisms: An experience with snakes. J Herpetol. 2012;46: 423–428. doi: 10.1670/11-118

[44] Crane E. The World History of Beekeeping and Honey Hunting. New York: Routledge; 1999.

[45] Hill RJ, Osgood CE, Suci GJ, Tannenbaum PH. The Measurement of Meaning. Am Sociol Rev. 1958;23: 227 doi: 10.2307/2089024

[46] Drissner J, Haase H, Rinderknecht A, Hille K. Effective environmental education through half-day teaching programmes outside school. ISRN Educ. 2013;2013: 1–6. doi: 10.1155/2013/503214

[47] Field A. Discovering Statistics using IBM SPSS Statistics [Internet]. Discovering Statistics using IBM SPSS Statistics. SAGE Publications, Ltd; 2013 Available: http://www.statisticshell.com/docs/cluster.pdf

[48] Bender R, Lange S. Adjusting for multiple testing—when and how? J Clin Epidemiol. 2001;54: 343–349. doi: 10.1016/S0895-4356(00)00314-0 11297884 10.1016/s0895-4356(00)00314-0

[49] Cohen J. A power primer. Psychol Bull. 1992;112: 155–159. doi: 10.1037/0033-2909.112.1.155 19565683 10.1037//0033-2909.112.1.155

[50] Mayring P. Qualitative Inhaltsanalyse [Qualitative Content Analysis]. Handb Qual Forsch der Psychol. 2010;1: 144 doi: 10.1007/978-3-8349-9441-7

[51] Cohen J. A coefficient of agreement of nominal scales. Educ Psychol Meas. 1960;20: 37–46. doi: 10.1177/001316446002000104

[52] Landis JR, Koch GG. The measurement of observer agreement for categorical data. Biometrics. 1977;33: 159–174. doi: 10.2307/2529310 843571

[53] Hutcheson G, Sofroniou N. The Multivariate Social Scientist [Internet]. Introductory statistics using generalized linear models. 6 Bonhill Street, London EC2A 4PU: SAGE Publications, Ltd.; 1999 doi: 10.4135/9780857028075

[54] Mingo J. Bees Make the Best Pets: All the Buzz About Being Resilient, Collaborative, Industrious, Generous, and Sweet–Straight from the Hive Conari Press; 2013.

[55] Prokop P, Fančovičová J. Tolerance of amphibians in Slovakian people: A comparison of pond owners and non-owners. Anthrozoos A Multidiscip J Interact People Anim. 2012;25: 277–288. doi: 10.2752/175303712X13403555186136

[56] Lindemann-Matthies P. “Loveable” mammals and “lifeless” plants: how children’s interest in common local organisms can be enhanced through observation of nature. Int J Sci Educ. 2005;27: 655–677. doi: 10.1080/09500690500038116

[57] Prokop P, Prokop M, Tunnicliffe SD. Is biology boring? Student attitudes toward biology. J Biol Educ. 2007;42: 36–39. doi: 10.1080/00219266.2007.9656105

[58] Prokop P, Tunnicliffe SD. Effects of Having Pets at Home on Children’s Attitudes toward Popular and Unpopular Animals. Anthrozoos A Multidiscip J Interact People Anim. 2010;23: 21–35. doi: 10.2752/175303710X12627079939107

[59] Liefländer AK, Fröhlich G, Bogner FX, Schultz PW. Promoting connectedness with nature through environmental education. Environ Educ Res. 2013;19: 370–384. doi: 10.1080/13504622.2012.697545

[60] Liefländer AK, Bogner FX. The Effects of Children’s Age and Sex on Acquiring Pro-Environmental Attitudes Through Environmental Education. J Environ Educ. 2014;45: 105–117. doi: 10.1080/00958964.2013.875511

[61] Münstedt K, Mühlhans A. Fears, phobias and disgust related to bees and other arthropods. Adv Stud Med Sci. 2013;1: 125–142. Available: http://m-hikari.com/asms/asms2013/asms1-4-2013/muenstedtASMS1-4-2013.pdf

[62] Flavell JH. Perspectives on perspective taking. Piaget’s theory: Prospects and Possibilities. 1992 pp. 107–139.

[63] Bogner FX, Wilhelm MG. Environmental perspectives of pupils: the development of an attitude and behaviour scale. Environmentalist. 1996;110: 95–110. doi: 10.1007/BF01325101

[64] Leach J, Driver R, Scott P, Wood‐Robinson C. Children’s ideas about ecology 2: ideas found in children aged 5–16 about the cycling of matter. Int J Sci Educ. 1996;18: 19–34. doi: 10.1080/0950069960180102

[65] Leach J, Driver R, Scott P, Wood‐Robinson C. Children’s ideas about ecology 3: ideas found in children aged 5–16 about the interdependency of organisms. Int J Sci Educ. 1996;18: 129–141. doi: 10.1080/0950069960180201

[66] Çalik M, Ayas A. A comparison of level of understanding of eighth-grade students and science student teachers related to selected chemistry concepts. J Res Sci Teach. 2005;42: 638–667. doi: 10.1002/tea.20076

[67] Tautz J, Heilmann HR. Phänomen Honigbiene [Phenomenon Honeybee] München: Elsevier Spektrum Akademischer Verlag; 2007.

[68] Killermann W. Biology education in Germany: Research into the effectiveness of different teaching methods. Int J Sci Educ. 1996;18: 333–346. doi: 10.1080/0950069960180306

[69] Nisbet EK, Zelenski JM, Murphy SA. Linking individuals’ connection with nature to environmental concern and behavior. Environ Behav. 2009;41: 715–740. doi: 10.1177/0013916508318748

[70] Schlegel J, Breuer G, Rupf R. Local insects as flagship species to promote nature conservation? A survey among primary school children on their attitudes toward invertebrates. Anthrozoos A Multidiscip J Interact People Anim. 2015;28: 229–245. doi: 10.2752/089279315X14219211661732

[71] Gupta RK. Beekeeping for Poverty Alleviation and Livelihood Security [Internet]. Gupta RK, Reybroeck W, van Veen JW, Gupta A, editors. Beekeeping for Poverty Alleviation and Livelihood Security. Dordrecht: Springer Netherlands; 2014 doi: 10.1007/978-94-017-9199-1

