# Supplementary material for: Individual perception of bees: Between perceived danger and willingness to protect
Source: PLoS One. 2017 Jun 29;12(6):e0180168. doi: 10.1371/journal.pone.0180168 (PMC5491143; doi:10.1371/journal.pone.0180168)
Supplement: S1 Table — (DOCX) [file pone.0180168.s001.docx]

**S1 Table:** Inductively built categories for the open question “Explain why bees are supposed to be dangerous / safe in your opinion?”.

| **Category** | **Description** | **Example** |
| --- | --- | --- |
| *Character of bees* |  |  |
| Breed | Argumentation refers to the breeding of a peaceful race (e.g. *Apis mellifera carnica*) | “Due to breeding, bees are safe” |
| Behavior of the bees | Argumentation refers to the behavior and character traits of bees. It is argued from the bees’ perspective. Intentions, instincts and characteristics, which cause a bee’s behavior, are also included. | “Bees just defend their bee colony, otherwise they are safe” |
| *Health aspects* |  |  |
| Bee sting | Argumentation includes an explicit naming of the bee sting with or without a valuation. | “The sting is the last consequence” |
| Unproblematic | Argumentation includes a value judgement like the sting is unproblematic because it is rare, painless, etc. | “The amount of poison is low, the pain goes away” |
| Problematic | Argumentation includes a value judgement like the sting is problematic because it is unpleasant, painful, etc. | “Stings can be very unpleasant” |
| Allergy | Argumentation includes the aspect that a sting could be dangerous for a person with a bee venom allergy. | “Bees only pose a risk for a person with a bee venom allergy” |
| *Handling of bees* |  |  |
| Appropriate human behavior | Argumentation refers to active human behavior regarding the handling of bees. It is argued from the humans’ perspective. | “Bees are harmless If you don’t provoke them” |
| *Other* |  |  |
| Other | Any other reasons. | “Bees are the third most important animal used for production” |
