# Supplementary material for: Individual perception of bees: Between perceived danger and willingness to protect
Source: PLoS One. 2017 Jun 29;12(6):e0180168. doi: 10.1371/journal.pone.0180168 (PMC5491143; doi:10.1371/journal.pone.0180168)
Supplement: S2 Table — (DOCX) [file pone.0180168.s002.docx]

**S2 Table.** Inductively built categories for the open question “Explain why bees are supposed to be worth protecting / worthless in your opinion?”.

| **Category** | **Description** | **Example** |
| --- | --- | --- |
| *Achievements of bees for man & nature* |  |  |
| Pollination | Pollination is explicitly or implicitly mentioned with or without any further specifications | “There would be few fruits without bees” |
| Human importance | Pollination service is mentioned in context with the importance for humans. It includes the pollination of crop plants and fruits, food security, etc. | “Bees ensure that we have nutrition (pollination)” |
| Ecological importance | Pollination service is mentioned in context with the importance for the ecosystem. It includes general statements about the importance for nature as well as concrete statements about the conservation of an ecological balance, etc. | “The pollination service of bees is essential for the most important wild and cultivated plants” |
| Bee products | Direct products of the honeybees are mentioned, e.g. honey, Propolis, wax, etc. | “Bees are the supplier of important food products like honey or royal jelly” |
| Extinction of humanity | It is mentioned that humanity would die out if bees died out, often referred to an allegedly quote of Albert Einstein. This category also includes argumentations about a following reduction of oxygen. | “If bees die out mankind will follow 4 years later (according to A. Einstein)” |
| *Conservation* |  |  |
| Survival of the species | The survival of the bee species is mentioned explicitly or implicitly by conservation of biodiversity regarding the bee or by protecting a creature in general. | “Bees are basically worth to be protected because they are creatures” |
| *Other* |  |  |
| Unspecific arguments | Unspecific arguments about the usefulness and importance of the bees that often do not include any further explanations. | “Bees are worth to be protected because they are important for us” |
| Hobby & research | Keeping bees as hobby or the bee as object of study. | “You can learn a lot from them, for example social behavior” |
| Other | Any other reason. | “Insects rule the world” |
